# Timing the Scan: Optimizing Screening for Osteoporosis and Risk of Fracture in Celiac Disease

**DOI:** 10.14309/ajg.0000000000003750

**Published:** 2025-08-26

**Authors:** Francesco Tovoli, Guido Zavatta, Giovanni Monaco, Dante Pio Pallotta, Kinga Skoracka, Alberto Raiteri, Agnese Pratelli, Maria Boe, Iwona Krela-Kaźmierczak, Uberto Pagotto, Alessandro Granito

**Affiliations:** 1 Department of Medical and Surgical Sciences (DIMEC), University of Bologna, Bologna, Italy;; 2 Division of Internal Medicine, Hepatobiliary and Immunoallergic Diseases, IRCCS Azienda Ospedaliero-Universitaria di Bologna, Bologna, Italy;; 3 Division of Endocrinology and Diabetes Prevention and Care, IRCCS Azienda Ospedaliero-Universitaria Di Bologna, Bologna, Italy;; 4 Department of Gastroenterology, Dietetics and Internal Diseases, Poznan University of Medical Sciences, Poznan, Poland;; 5 Doctoral School, Poznan University of Medical Sciences, Poznan, Poland;; 6 Laboratory of Nutrigenetics, Department of Gastroenterology, Dietetics and Internal Diseases, Poznan University of Medical Sciences, Poznan, Poland.

## Abstract

**INTRODUCTION:**

Patients with celiac disease (CeD) have an increased risk of osteoporosis and fractures, but the ideal timing for bone mineral density (BMD) assessment remains unclear due to conflicting recommendations. This study evaluated the optimal timing for dual-energy x-ray absorptiometry (DXA) screening considering different clinical targets: early detection of BMD alterations, osteoporosis diagnosis, or fracture risk stratification.

**METHODS:**

Observational study prospectively enrolling 627 patients with CeD (>25 years) who underwent DXA scans of the lumbar spine and hip as part of standard care. Data on clinical presentation, serology, histology, and fracture risk were analyzed. Logistic regression identified risk factors of low BMD and osteoporosis. The reliability of the National Osteoporosis Guidelines Group (NOGG) guidelines for avoiding unnecessary DXAs was assessed.

**RESULTS:**

Low BMD for age was present in 17.2% of patients, with significant prevalence in the 25–34 years age group (13.4%), further increasing in the 45–54 years age group. Osteoporosis was detected in 17.9% of patients, with prevalence increasing significantly in patients aged older than 45 years. Risk factors included weight loss, underweight status, and iron-deficiency anemia. Using the NOGG criteria, 67% of patients could have avoided DXA, with a 0.5% risk of missing clinically significant findings requiring treatment (but also losing 15.7% patients with low BMD for age).

**DISCUSSION:**

The ideal timing for DXA screening in patients with CeD depends on the clinical objective. DXA at diagnosis maximizes the early detection of low bone mass, whereas the NOGG criteria effectively identified patients at high risk of fractures, reducing unnecessary scans. However, their use should be weighed against an underdetection of clinically relevant BMD alterations. Tailoring DXA timing to healthcare resources and patient demographics may optimize outcomes and resource allocation.

## Graphical abstract

**Figure:**
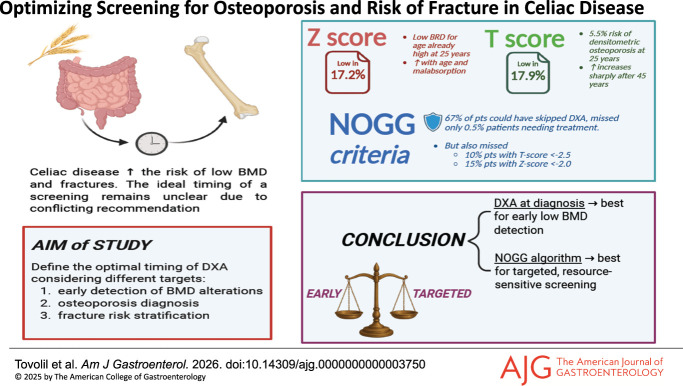

## INTRODUCTION

Metabolic bone disease is a common manifestation in patients with celiac disease (CeD). Although adherence to a gluten-free diet has been shown to improve bone mineral density (BMD), adult patients with CeD continue to face an exceedingly high fracture risk, estimated at 320–480 per 100,000 person-years (1–3). Furthermore, population-based studies indicate that fracture incidence remains comparable before and after CeD diagnosis (1), underscoring the need for early identification and proactive management of metabolic bone disease to prevent fragility fractures.

Despite the significance of this issue, there is no universal consensus on the optimal timing for screening metabolic bone disease in CeD. The American College of Gastroenterology (4) and the European Society for the Study of Coeliac Disease (5) recommend performing dual-energy x-ray absorptiometry (DXA) at diagnosis, with the latter guideline allowing postponement until the age of 35 years. Conversely, the British Society of Gastroenterology recommends DXA only in patients with risk factors of osteoporosis or those older than 55 years (6). Finally, the National Institute for Health and Care Excellence (NICE) guidelines for CeD (7) endorse the proposal of the National Osteoporosis Guidelines Group (NOGG) (8) and suggest a preliminary risk assessment using clinical scoring tools, with DXA reserved for individuals exceeding specific risk thresholds and monitoring limited to patients undergoing treatment.

These discrepancies might reflect different primary objectives and resource considerations when determining the ideal timing for BMD screening. One possible approach prioritizes the early detection of low BMD for age, which often represents the earliest alteration in young patients with secondary causes of metabolic bone disease. Although low BMD for age does not automatically warrant antiosteoporotic therapy, it frequently prompts other interventions, such as lifestyle interventions, dietary modifications, calcium and vitamin D supplementation (when appropriate), and closer clinical follow-up. Another approach might emphasize the early detection of densitometric osteoporosis, a condition strongly associated with an increased risk of fragility fractures (9,10). In some national healthcare systems, a diagnosis of osteoporosis is sufficient to recommend initiating treatment. Alternatively, screening could focus on identifying patients at an elevated risk of fragility fractures (as in the case of the NICE guidelines) regardless BMD. Fracture risk is influenced by BMD and additional factors, including age, sex, previous fragility fractures, family history of hip fractures, smoking, alcohol consumption, and corticosteroid use. Among the tools available for fracture risk assessment, the Fracture Risk Assessment Tool (FRAX) is the most validated and widely used, estimating the 10-year probability of major fragility fractures, with or without incorporating BMD measurements (11). In many healthcare systems, decisions regarding antiosteoporotic therapy rely more heavily on overall fracture risk than on BMD values alone.

The aim of this study was to address gaps in current practice by evaluating the utility and outcomes of DXA screening in patients with CeD, contextualizing our findings within the framework of the outlined scenarios. Through this approach, we seek to provide evidence to inform guidelines and enhance the management of metabolic bone disease in this population.

## METHODS

### Design of the study

This study is part of a broader project, Celiac Disease Manifestations and Complications, designed to prospectively evaluate the clinical presentation and long-term outcomes of patients with CeD. Patients are enrolled at the time of CeD diagnosis, and their data are systematically recorded in a prospectively maintained database.

For the purpose of this study, patients aged older than 25 years with a new diagnosis of CeD were prospectively enrolled and prescribed DXA scans of the lumbar spine and hip (January 2014–October 2024) as part of standard clinical practice for CeD, in accordance with local guidelines (12). Patients were advised to complete the examination within 1 year from the diagnosis. DXA findings were integrated with clinical data, serology, and histology. For analysis, patient age was categorized into the following groups: 25–34, 35–44, 45–54, 55–64, and >65 years. The prevalence and risk factors of low BMD and osteoporosis were calculated. Finally, we assessed the reliability of an approach based on performing DXA only in patients exceeding previously proposed age-driven fracture risk thresholds (8).

### Clinical data

Collected data included patient demographics (age at diagnosis, sex, body weight, height), clinical presentation (signs and symptoms), and serological results. Medical records also systematically documented family history of fragility fractures, smoking and alcohol habits, history of previous fractures, concurrent illnesses, and ongoing medications.

### Diagnosis of CeD

CeD was diagnosed by serological testing of CeD-specific antibodies (antitransglutaminase immunoglobulin [Ig] A or IgG in patients with IgA deficiency) and confirmed by duodenal mucosal biopsies in all patients (4). Histological examination results were reported according to the Marsh criteria modified by Oberhuber (13). Patients with seronegative or potential CeD were included in the study, under strict diagnostic criteria. According to the Oslo definitions and established international guidelines, seronegative CeD was identified by the presence of gluten-related symptoms, negative serology, human leukocyte antigen-DQ2/DQ8 positivity, villous atrophy, exclusion of other causes of duodenal mucosal flattening, and clinical-histological remission after 12 months on a gluten-free diet (4,5,14). Potential CeD was defined by persistent serological positivity for both antitransglutaminase IgA and antiendomysium IgA, confirmed by 2 determinations at least 6 weeks apart, presence of human leukocyte antigen-DQ2/DQ8 and absence of histological abnormalities (4,5,14).

### DXA prescription

DXA was prescribed as part of standard clinical practice at the time of CeD diagnosis (12). Patients were encouraged to perform DXA scans within the Radiology Unit of our institution to minimize variability, although external scans were permitted when institutional slots were unavailable. In the latter cases, we considered only scans performed in sites where quality assurance measures, such as calibration of DXA machines, were in place.

### DXA parameters

BMD values were reported as *T*-scores and *Z*-scores. *T*-scores represent deviations from the mean BMD of a young adult reference population, whereas *Z*-scores compare values to an age-matched and sex-matched reference population. Osteoporosis (*T*-score ≤−2.5), and BMD below or within the expected range for age (*Z*-score ≤ or >−2.0, respectively) were classified according to the World Health Organization and International Society for Clinical Densitometry criteria (15,16). DXA scans were conducted and analyzed in adherence to manufacturer protocols (17).

### Risk of fracture

Fracture risk was estimated using the Fracture Risk Assessment Tool (FRAX) for the Italian population (available at https://www.sheffield.ac.uk/FRAX/tool.aspx?lang=en) (11). The FRAX tool has already been validated as a reliable predictor of fragility fractures, including in the specific context of CeD (18,19). For patients younger than 40 years, the calculator defaulted to a minimum age of 40 years. CeD was considered a cause of secondary osteoporosis in FRAX calculations to enhance the predictive accuracy of fracture risk (19).

### Statistics

To clarify the utility of DXA for the early detection of low BMD for age, we reported the prevalence of this condition across decades as frequencies and investigated the factors associated with low BMD for age using a binary logistic regression in which age, sex, symptoms potentially attributable to malabsorption (diarrhea, underweight, weight loss, iron deficiency anemia), serology (anti-tissue transglutaminase IgA antibodies >10× the upper normal limit vs controls), and histology (Marsh 3a–3c vs 1–2) were independent variables. Variables with a *P* value <of 0.10 were included in the multivariable model. The role of DXA in detecting densitometric osteoporosis was similarly assessed, using *T*-scores (≤−2.5 vs >−2.5) as the dependent variable.

Since *Z*-score can underestimate the proportion of older patients with low BMD and *T*-score might underestimate BMD loss at a younger age, we performed an additional analysis using a composite end point termed “clinically relevant BMD alterations,” defined as a *Z*-score ≤−2.0 in men aged younger than 50 years and premenopausal women, or a *T*-score ≤−2.5 in men aged 50 years or older and postmenopausal women. These criteria reflect clinical practice and guideline recommendations from the World Health Organization and International Society for Clinical Densitometry (15,20).

The applicability of the NICE-NOGG guideline approach was evaluated by determining the rate of patients who could have theoretically avoided an initial DXA (as they did not reach the lower assessment threshold) but presented BMD values so impaired that the post-DXA FRAX score exceeded the treatment threshold. In brief, according to the NICE-NOGG recommendations, the lower assessment thresholds for the probability of a major osteoporotic fracture were 2.6%, 2.7%, 3.4%, 4.5%, 5.9%, 8.4%, and 11% for patients aged 40, 45, 50, 55, 60, 65, and older than 70 years, respectively (8). The intervention thresholds for treatment were 5.9%, 6.0%, 7.2%, 9.4%, 12%, 16%, and 20% for the same age groups (8). For this analysis, patients aged younger than 40 years at the time of CeD diagnosis were categorized as individuals for whom DXA assessment was not indicated under the NOGG guidelines (8). However, they were evaluated using the same intervention threshold as patients aged 40–44 years to maintain consistency.

A *P* value of < 0.05 was considered statistically significant for all the analyses. Statistical analysis was performed using STATA 18SE (StataCorp, Lakeway, TX).

### Ethics

This study was approved by the Institutional Review Board of the Bologna Authority S. Orsola-Malpighi Hospital (Protocol 243/2013/O/OssN) and was subsequently renotified and approved after the integration of the previous institution into the newly established IRCCS Azienda Ospedaliero-Universitaria di Bologna (Protocol 427/2021/Oss/AOUBo). The study was conducted in accordance with the guidelines of the Declaration of Helsinki. Informed consent was obtained in compliance with Institutional Review Board requirements.

## RESULTS

### Study population

During the time frame of the study, 687 patients satisfying the inclusion criteria were included and prescribed a DXA. Of them, 60 (8.7%) did not perform the DXA within the specified time range and were therefore excluded from the study. Consequently, the final study population consisted of 627 patients.

Most patients were female (78%), with a mean age of 42.0 ± 12.6 years (Table 1). Seronegative (n = 12, 1.9%) and potential CeD (n = 30, 4.8%) accounted only for a slim minority of the population.

**Table 1.**
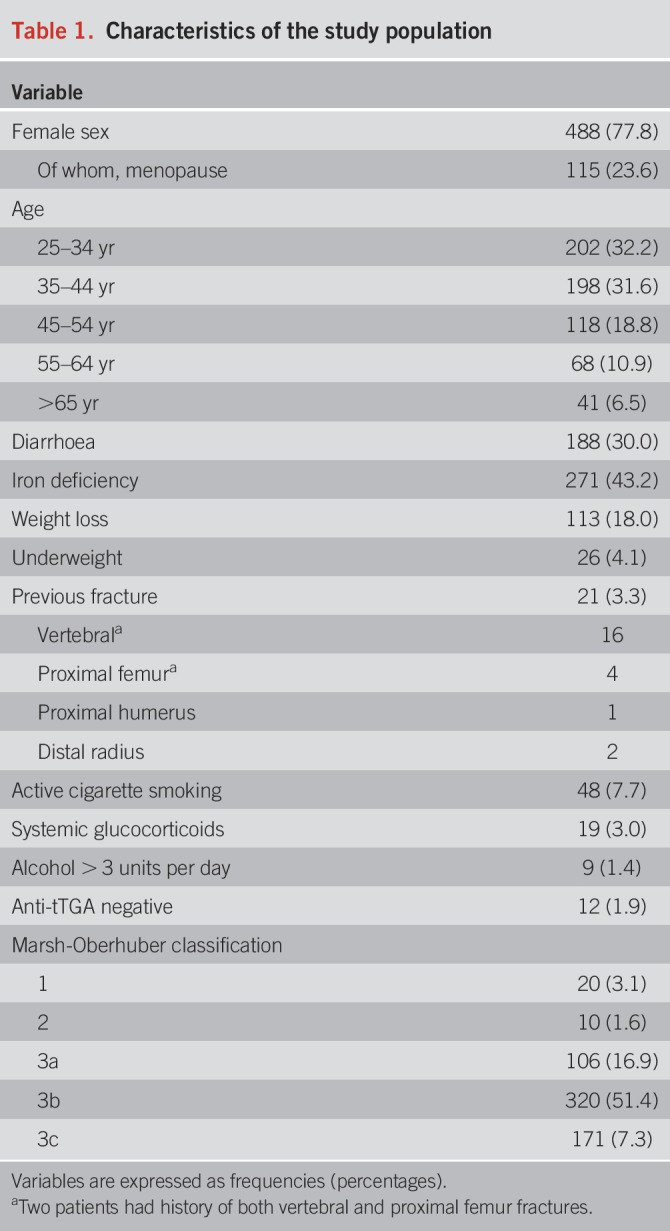
Characteristics of the study population

### Prevalence and risk factors for low BMD for age

**Figure 1.**
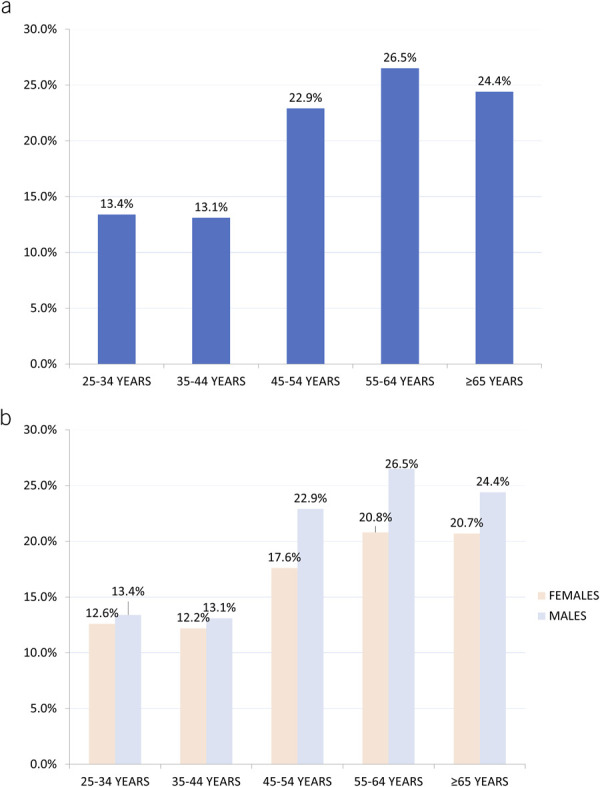
Prevalence of low bone mineral density for age (*Z*-score ≤−2) stratified for age (**a**) and for age and sex (**b**).

One hundred eight patients (17.2%) had low BMD for age in the whole study population. The prevalence of low BMD for age was already relevant in the 25–34 years age group (13.4%), significantly increasing in the 45–54 years age group (22.9%), and then stabilizing with a lack of significance in the >65 years age group, likely due to the small sample size in this subgroup (Figure 1). Additional risk factors of low BMD for age included weight loss, underweight status, and iron-deficiency anemia (Table 2). We found no differences in the prevalence of low BMD for age in patients who underwent the DXA in our institution and those evaluated in other centers (89/520 [17.1%] vs 19/107 [17.8%], *P* = 0.871).

**Table 2.**
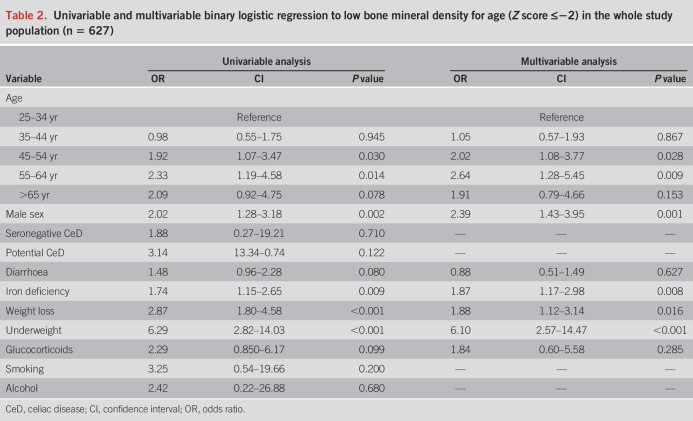
Univariable and multivariable binary logistic regression to low bone mineral density for age (*Z* score ≤−2) in the whole study population (n = 627)

### Prevalence and risk factors for osteoporosis

**Figure 2.**
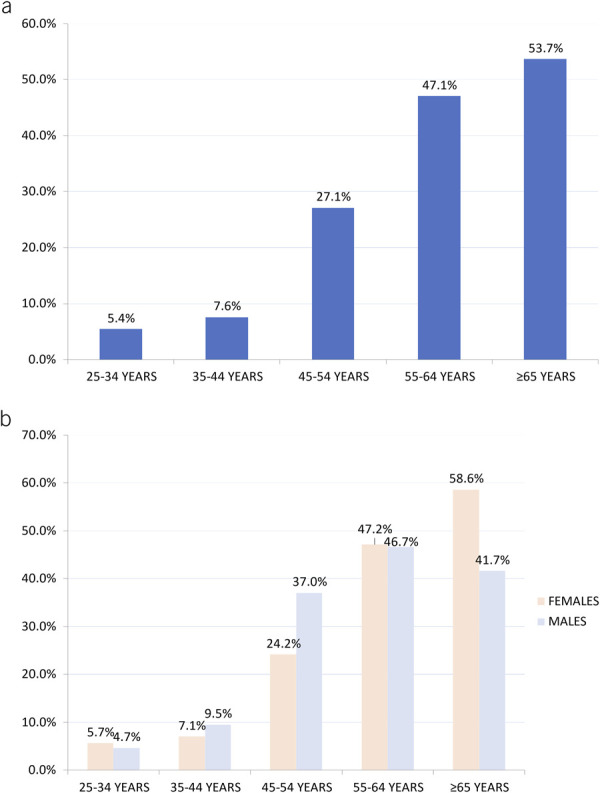
Prevalence of osteoporosis (*T*-score ≤−2.5) stratified for age (**a**) and for age and sex (**b**).

One hundred twelve patients (17.9%) had densitometric osteoporosis in the whole study population. The prevalence of osteoporosis was relatively low in the 25–34 and 35–44 years age groups (5.5% and 7.6%, respectively), with a clinical and statistically significant surge in the following decades (Figure 2). Other risk factors of densitometric osteoporosis included symptoms of malabsorption at the diagnosis of CeD, such as weight loss and underweight status. Conversely, female sex was not associated with an increased risk of *T*-score <−2.5 (Table 3). Among the 515 patients without osteoporosis, 29 (5.6%) had low BMD for age. We found no differences in the prevalence of osteoporosis in patients who underwent the DXA in our institution and those evaluated in other centers (92/520 [17.7%] vs 20/107 [18.7%], *P* = 0.806).

**Table 3.**
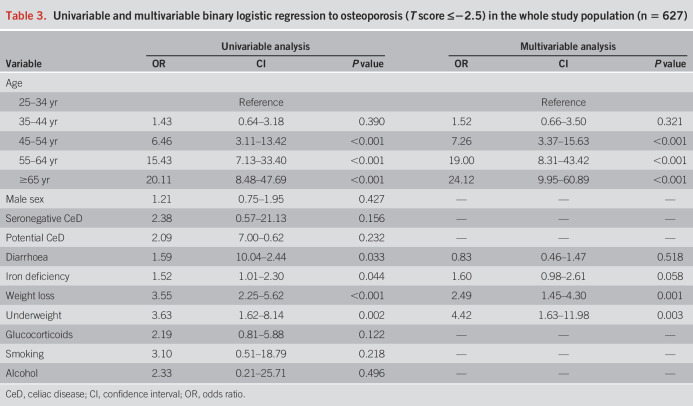
Univariable and multivariable binary logistic regression to osteoporosis (*T* score ≤−2.5) in the whole study population (n = 627)

### Prevalence and risk factors for clinically relevant BMD alterations

**Figure 3.**
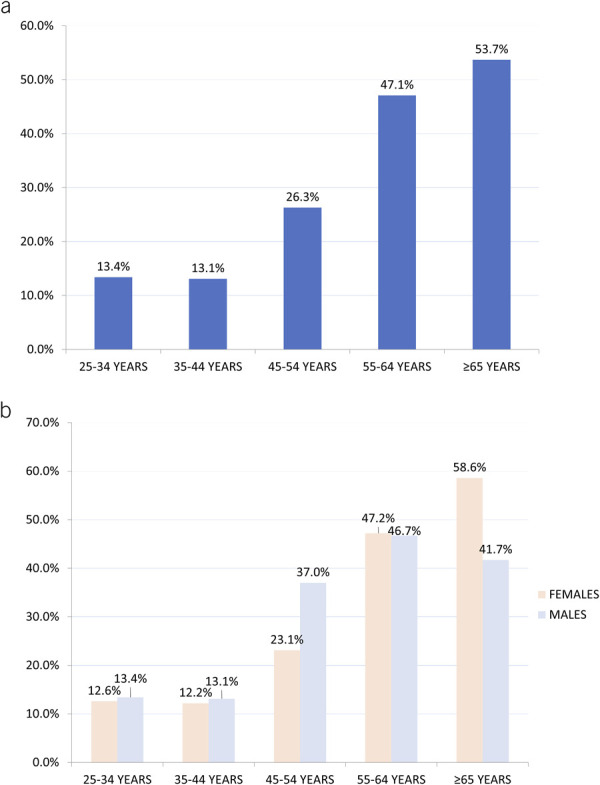
Prevalence of clinically relevant bone mineral density alterations (*Z*-score ≤−2.0 in men aged younger than 50 years and premenopausal women, or *T*-score ≤−2.5 in men aged 50 years or older and postmenopausal women) stratified for age (**a**) and for age and sex (**b**).

One hundred thirty-eight patients (22.0%) had clinically relevant BMD alterations (Figure 3). The factors associated with this composite end point were age group, weight loss, and underweight status (Supplementary Table, Supplementary Digital Content 1, http://links.lww.com/AJG/D774).

### Risk of fracture and reliability of the NICE-NOGG proposal

Overall, the 10-year risk of major fragility fractures exceeded the age-specific threshold for prescribing DXA according to the NOGG guidelines in 206 patients (32.9%). Among the 421 patients below the assessment threshold, 66 (15.7%) and 42 (10.0%) had low BMD for age and osteoporosis, respectively. Sixty-eight patients (16.2%) were below the assessment threshold while having clinically relevant BMD alterations, according to the criteria used to define this end point.

**Figure 4.**
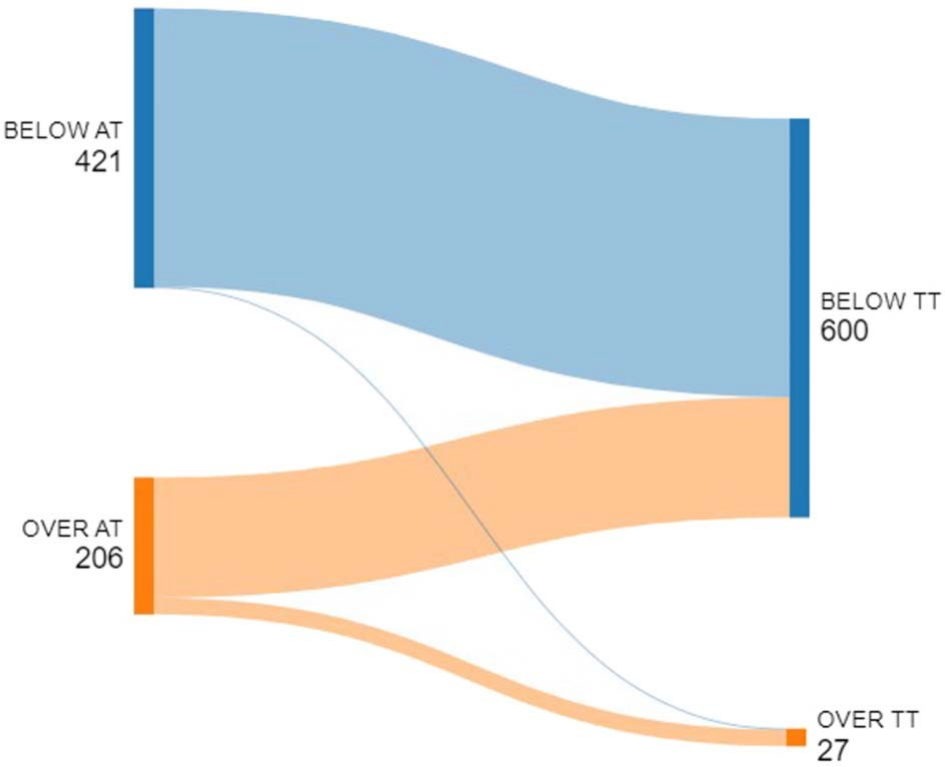
Classification of the study population according to the National Osteoporosis Guidelines Group guidelines. On the left, proportion of patients below and over the age-specific lower assessment threshold (AT) according to the Fracture Risk Assessment Tool (FRAX) score calculated without bone mineral density information. On the right, recategorization of patients as below and over the age-specific treatment threshold (TT) after considering bone mineral density in the calculation of the FRAX score.

All enrolled patients underwent DXA because of local policies, regardless of their pre-DXA risk assessment. After recalculating the FRAX score with BMD values, 27 patients (4.3%) exceeded the age-specific threshold for intervention. Among these, 2 patients had a pre-BMD risk below the threshold for prescribing DXA. Consequently, the prevalence of patients exceeding the intervention threshold was 0.5% among those initially classified as low-risk based on pre-DXA calculations and 12.1% in the remaining population (Figure 4).

## DISCUSSION

Metabolic bone disease is a significant and relatively underevaluated manifestation of CeD, with discordant recommendations for its detection and management from different international guidelines (21). In this real-life study, we evaluated the optimal timing for the first DXA scan depending on the desired target: (i) early detection of low BMD for age, (ii) densitometric osteoporosis, or (iii) identification of patients at high-fracture risk only.

Low BMD for age was already high in the 25–34 years age group (13.4%), with a further increase in subsequent decades and in patients with features of malabsorption. In a scenario with sufficient resources allowing for a very early diagnosis of BMD alterations as the target, the ideal timing for the first DXA scan would be at the time of CeD diagnosis. This proposal would be supported by the fact that the 25–34 years age group already had a significant prevalence of low BMD for age (based on the *Z*-score definition, the prevalence of patients with values <−2.0 SDs should, in fact, be <2.3% in the general population of the same age and sex). This scenario aligns with the recommendations of the American College of Gastroenterology guidelines (4). It is worth noting that a portion of the expenses could be offset by adopting a more relaxed DXA follow-up protocol for patients without significant BMD alterations. This approach is supported by evidence showing that DXA parameters improve after initiating a gluten-free diet and remain largely stable even 7–10 years after CeD diagnosis (18,22).

By contrast, in a scenario with intermediate resource availability, the timing of the first DXA scan could be guided by the probability of diagnosing densitometric osteoporosis. In our study, the prevalence of osteoporosis was low in absolute terms (<10%) in the 25–44 years age group but increased significantly and rapidly in subsequent decades. Other risk factors included weight loss and underweight status. These findings are consistent with a recent large-scale study on patients with patients from the United Kingdom (18). Based on our results, the ideal timing would be at the age of 45 years or at the diagnosis of CeD if additional risk factors are present. This proposal is similar to the British Society of Gastroenterology recommendations, which, however, sets the threshold at the age of 55 years (6). Based on our study findings, a lower age threshold might appear more prudent, as the prevalence of osteoporosis in the 45–54 years age group was not negligible (27.1%).

Finally, in a scenario focused exclusively on evaluating the 10-year fracture risk, the NICE-NOGG algorithm demonstrated significant effectiveness in identifying a large group of patients for whom performing DXA would not provide additional clinically meaningful information. Specifically, approximately 67% of patients could have avoided DXA, with only a 0.5% risk of missing BMD values that would confer a clinically significant increase in fracture risk warranting consideration for antiosteoporotic treatment. These findings validate recent studies reporting minimal differences between fracture risk assessments performed with or without BMD values in patients with CeD (18,23), probably due to the relatively young age of the included patients. However, it is necessary to note that 15.7% and 10.0% of patients with a FRAX score below the threshold had retrospectively low BMD for age and osteoporosis, resulting in 16.2% patients having clinically relevant BMD alterations. Moreover, further caution is warranted in the universal application of the NOGG algorithm for different reasons. First, no studies have specifically evaluated whether delayed DXA testing negatively affects the extent of bone mass recovery, and 1 large population-based study found a comparable risk of fractures before and after gluten-free diet initiation (1). This uncertainty cannot be resolved by our study, as DXA was systematically performed at diagnosis in accordance with local guidelines. Second, knowledge of low BMD at diagnosis may enhance adherence to the gluten-free diet, particularly in asymptomatic or minimally symptomatic patients because of its well-known beneficial effect on BMD recovery. Third, younger patients with low BMD may require individualized long-term monitoring beyond standard risk algorithms. Fourth, older patients—which were inevitably underrepresented in our cohort—may harbor risk factors such as falls or prolonged undiagnosed malabsorption that are not fully captured by FRAX, potentially leading to an underestimation of their fracture risk. Finally, since the NOGG algorithm was developed in the UK context, its applicability to healthcare systems with a more limited access to BMD testing remains uncertain. Some studies have explored the use of FRAX-based, age-dependent intervention thresholds in non-European settings, including Lebanon and several Latin American countries (24–26). While a different fracture risk epidemiology may require country-specific intervention thresholds, these studies support the underlying methodological framework adopted by NOGG and provide a minimal rationale for its broader applicability. Nonetheless, further validation in resource-constrained settings remains essential.

A key strength of this study is its evaluation of the prevalence and risk factors of both low BMD for age and osteoporosis in a cohort of patients with CeD from a country with universal healthcare access. This setting minimized selection bias. In addition, prospective enrollment and a low rate of noncompliance with DXA prescriptions further reduced the risk of selection bias. To our knowledge, this is the largest study to assess the prevalence of metabolic bone disease and the role of the FRAX score in patients with CeD. However, some limitations should be acknowledged. First, this study was conducted at a single tertiary center in Italy, which may limit the generalizability of the results to other populations. Second, the assessment of trabecular bone score, parathyroid hormone, inflammatory markers, and bone turnover biomarkers was not performed systematically as they are not part of the panel of recommend for CeD at the diagnosis. These parameters may help refine risk stratification in CeD and could be considered in future research, given the distinctive pathophysiology of metabolic bone disease in this population. Third, some data, such as smoking status and alcohol intake, were self-reported, introducing the potential for reporting bias, though this limitation reflects routine clinical practice. It is also worth noting that FRAX, although an extremely valuable and user-friendly tool, has some limitations. The calculator defaults to a minimum age of 40 years; moreover, it does not take into account interactions between several clinical risk factors. On the other hand, the FRAX score is country-dependent and can be used in different populations. Perhaps, given the incidence of secondary osteoporosis, it would be worthwhile to consider its refinement to enhance its precision and relevance. In particular, future studies might explore the use of alternative scores or tools designed to capture the specific fracture risk in younger patients with CeD. Finally, not all patients underwent DXA with the same machine. However, BMD values from different DXA machines are known to be highly correlated (27), and this limitation is also inherent in real-world clinical settings.

In conclusion, our findings highlight the importance of tailoring the timing of DXA screening to the intended clinical target and healthcare system in patients with CeD. For the early detection of bone loss, performing DXA at the time of CeD diagnosis is the most effective approach. Conversely, for a more resource-conscious strategy focused on reducing the 10-year fracture risk, the NOGG algorithm provided a robust framework to avoid unnecessary DXAs in approximately two-thirds of patients, with minimal risk of missing clinically significant cases. These results underscore the tradeoff between a proactive, standardized management strategy aimed at early detection and a cost-effective, targeted approach that reduces patient burden but may delay diagnoses in some cases. The choice between the different strategies depends on geographical and healthcare factors. For example, the first strategy may be more suitable in regions with a high prevalence of low BMD and abundant healthcare resources, whereas the second strategy might provide a better balance in settings with a low pretest probability of low BMD and/or limited resources. By presenting these findings, we aim to inform future guidelines, contextualizing the results within specific healthcare settings.

## CONFLICTS OF INTEREST

**Guarantor of the article:** Francesco Tovoli, MD.

**Specific author contributions:** F.T.: planned the study, analyzed data, drafted the manuscript. G.Z.: planned the study interpreted data. G.M.: collected data. D.P.P.: collected data. K.S.: interpreted data. A.R.: collected and interpreted data. A.P.: collected data. M.B.: collected data. I.K.: interpreted data. U.P.: interpreted data. A.G.: analyzed and interpreted data. All authors approved the final draft submitted.

**Financial support:** None to report.

**Potential competing interests:** None to report.

**Study Highlights**

**WHAT IS KNOWN**

✓ Patients with celiac disease have an increased risk of osteoporosis and fractures.
✓ The optimal timing for the first bone mineral density (BMD) assessment is uncertain.

**WHAT IS NEW HERE**

✓ Low BMD is highly prevalent, even among young patients.
✓ Osteoporosis is associated with clinical presentation and age >45 years.
✓ High-risk fracture patients are less common and can be identified using National Osteoporosis Guidelines Group criteria.
✓ The timing of BMD assessment should be guided by clinical objectives and resource availability.

## Supplementary Material

## ABBREVIATIONS:

AT: assessment threshold
BMD: bone mineral density
CeD: celiac disease
CI: confidence interval
DXA: dual-energy x-ray absorptiometry
FRAX: Fracture Risk Assessment Tool
Ig: immunoglobulin
NICE: National Institute for Health and Care Excellence
NOGG: National Osteoporosis Guidelines Group
OR: odds ratio
SD: standard deviations
TT: treatment threshold
