# Supplementary material for: Timing the Scan: Optimizing Screening for Osteoporosis and Risk of Fracture in Celiac Disease
Source: Am J Gastroenterol. 2025 Aug 26;121(7):1752–61. doi: 10.14309/ajg.0000000000003750 (PMC13313486; doi:10.14309/ajg.0000000000003750)
Supplement: Supplementary file 1 [file acg-121-1752-s001.docx]

SUPPLEMENTARY TABLE: Univariable and multivariable binary logistic regression to clinically relevant bone mineral density alterations (Z-score ≤-2.0 in men <50 years and premenopausal women, or T-score ≤-2.5 in men ≥50 years and postmenopausal women) in the whole study population (n=627).

| UNIVARIABLE ANALYSIS | | | VARIABLE | MULTIVARIABLE ANALYSIS | | |
| --- | --- | --- | --- | --- | --- | --- |
| *OR* | *CI* | *p* | *Age* | *OR* | *CI* | *p* |
|  | Reference |  | 25-34 years |  | Reference |  |
| 0.98 | 0.55-1.75 | 0.945 | 35-44 years | 1.05 | 0.58-1.90 | 0.881 |
| 2.31 | 1.30-4.11 | 0.004 | 45-54 years | 2.41 | 1.32-4.38 | 0.004 |
| 5.76 | 3.08-10.77 | <0.001 | 55-64 years | 6.05 | 3.15-11.62 | <0.001 |
| 7.51 | 3.60-15.66 | <0.001 | ≥65 years | 7.28 | 3.38-15.71 | <0.001 |
| 1.32 | 0.85-2.05 | 0.211 | Male sex | - | - | - |
| 2.29 | 0.54-19.47 | 0.198 | Seronegative CeD |  |  |  |
| 1.96 | 0.67-5.70 | 0.217 | Potential CeD | - | - | - |
| 1.51 | 1.01-2.24 | 0.044 | Diarrhoea | 0.89 | 0.54-1.46 | 0.636 |
| 1.42 | 0.97-2.08 | 0.069 | Iron deficiency | 1.38 | 0.90-2.09 | 0.144 |
| 3.15 | 2.03-4.88 | <0.001 | Weight loss | 2.43 | 1.51-43.93 | <0.001 |
| 4.49 | 2.03-9.95 | <0.001 | Underweight | 4.67 | 1.93-11.31 | 0.001 |
| 2.12 | 0.82-5.50 | 0.121 | Glucocorticoids | - | - | - |
| 3.11 | 0.50-19.12 | 0.224 | Smoking | - | - | - |
| 1.78 | 0.16-19.75 | 0.640 | Alcohol | - | - | - |
